# Sarcopenic obesity is associated with coffee intake in elderly Koreans

**DOI:** 10.3389/fpubh.2023.990029

**Published:** 2023-02-02

**Authors:** Do-Youn Lee, Sunghoon Shin

**Affiliations:** ^1^Research Institute of Human Ecology, Yeungnam University, Gyeongsan-si, Gyeongbuk, Republic of Korea; ^2^Neuromuscular Control Laboratory, Yeungnam University, Gyeongsan-si, Gyeongbuk, Republic of Korea

**Keywords:** sarcopenic obesity, sarcopenia, obesity, coffee, elderly

## Abstract

This study assessed the association between sarcopenic obesity (S+O+) and coffee intake inelderly Koreans. This study obtained data from the Korea National Health and Nutrition Examination Survey (KNHANES, 2008–2011), a cross-sectional and nationally representative survey conducted by the Korean Centers for Disease Control and Prevention. Of the 2,661 participants included in this study, there was a significant difference between 5.861 (95% CI 2.024–16.971) in less than one cup of coffee, and 6.245 (95% CI 2.136–18.260) in one cup of coffee, and 4.323 (95% CI 1.457–12.824) in two cups of coffee compared to three or more than cups of coffee. In contrast, in the case of sarcopenia or obesity only (S+O- or S-O+), no significant difference was found in any model. The results suggest that the elderly who consume less than one cup of coffee per day had a greater risk of S+O+ than those who consume more than three cups per day. Furthermore, there was an association between coffee intake and sarcopenia but not with obesity. Therefore, coffee intake may have prevented musculoskeletal loss in these patients.

## 1. Introduction

Sarcopenia is a condition in which aging causes the loss of muscle mass, muscle strength, and physical performance (1). Moreover, loss of muscle mass is associated with metabolic disorders, osteoporosis, cardiovascular disease, physical impairment, and high mortality (2–5). Obesity is another aging-related health problem that increases the risk of sarcopenia (6). Sarcopenia and obesity can coexist, and sarcopenic obesity (S+O+) is a novel type of obesity characterized by high adiposity (fat accumulation) and low muscle mass (7). Older people with S+O+ are significantly more likely to have physical function deterioration and comorbidities than those with sarcopenia or obesity alone (8, 9).

Coffee is one of the most popular beverages in the world and consists of a variety of minerals such as caffeine and phenolic compounds, which have antioxidant and anti-inflammatory effects (10–12). The advantages of these coffee components are associated with the reduction in various diseases and mortality rates, such as diabetes, stroke, Parkinson's disease, and cancer (13–16). In addition, habitual coffee consumption has been reported to improve physical activity (17) and reduce the risk of falls in the elderly (18–20).

Previous studies have reported various results regarding the association between coffee intake and sarcopenia and obesity. In one study, habitual coffee intake of middle-aged people in Japan was positively correlated with skeletal muscle mass (19). In addition, *in vivo* coffee treatment may have a beneficial effect on the prevention of aging-related sarcopenia by increasing muscle weight, muscle strength, and muscle regeneration of old mice (20). Regarding its association with obesity, high coffee consumption has been suggested to be related to a low risk of abdominal obesity and reduced adiposity (21, 22). In contrast, some studies have suggested that coffee intake is not associated with abdominal obesity (23–25).

There are many previous studies on the relationship between coffee intake and sarcopenia and obesity, but they are either not done on humans, or the results are different. In addition, there are few studies on the association between S+O+ and coffee intake. Furthermore, current research on coffee mainly focuses on the effects of coffee components on various organ systems, such as cardiovascular system (26–28), and relatively less interest in their association with skeletal muscles. Therefore, the purpose of this study was to investigate the association between sarcopenia, obesity and coffee intake, and the association between S+O+ and coffee intake in the elderly in Korea.

## 2. Methods

### 2.1. Data source and sampling

**Figure 1:**
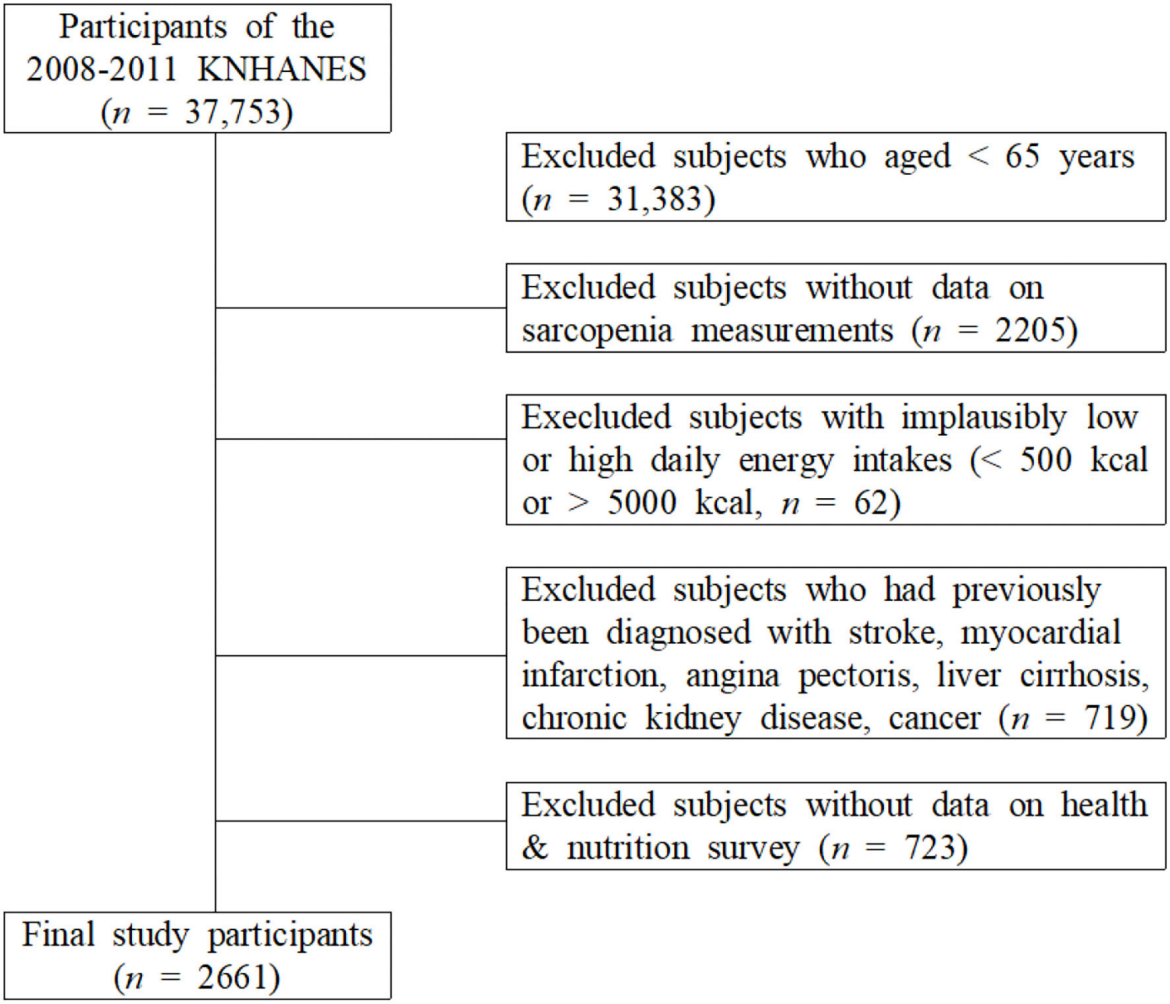
Selection of participants from the Korea National Health and Nutrition Examination Survey 2008–2011.

This study used data from KNHANES (2008–2011) conducted by the Korean Centers for Disease Control and Prevention. Those who responded to both the examination survey and the health survey among adults aged ≥ 65 years who underwent whole-body dual-energy X-ray absorptiometry (DXA) were included in this study. Among the 37,753 individuals who participated in the KNHANES, 6,370 individuals who were ≥ 65 years were selected. The following subjects were excluded: 62 subjects who reported implausibly low or high daily energy intakes (< 500 or > 5,000 kcal/day); 719 subjects who had previously been diagnosed with stroke, myocardial infarction, angina pectoris, liver cirrhosis, chronic kidney disease, and cancer; 2,205 individuals with sarcopenia; and 723 non-participants in the health and nutrition survey. Finally, 2,661 participants were selected (Figure 1).

### 2.2. Measurements of variables

#### 2.2.1. Covariates

Physical examination included height, weight, systolic and diastolic blood pressure (SBP, DBP), triglyceride, fasting glucose, body mass index (BMI), total cholesterol, and high-density lipoprotein cholesterol (HDL-C) measurement variables. Blood pressure was measured using a mercury sphygmomanometer in a seated position after a 10-min rest period. Two measurements were made for all subjects at 5-min intervals. The average of two measurements was used for the data analyses. Waist circumference (WC) was measured at the midpoint between the bottom of the rib cage and the top of the lateral border of the iliac crest during full expiration. Blood samples were collected from subjects in the morning after fasting overnight and analyzed at a national central laboratory. BMI was calculated by dividing the weight (kg) by the height (m^2^). Marital status was classified as living with or without a spouse. Individual income levels were divided into quartiles. For smoking status, subjects were categorized as non-smokers, ex-smokers, or current smokers, and for drinking status subjects were categorized as current users or non-users. The frequency of resistance exercise was assessed according to participants' answers to the question “How many times do you do resistance exercise (push-ups, sit-ups, lifting dumbbells or barbells) a week?” The short version of the IPAQ in Korea (29), which measures health-related physical activity in populations, was used to measure subjects' current walking. The number of days the subject walked ≥10 min at a time in the past week was expressed. Walking was measured by the total walking time in a week (TWT), calculated as follows: TWT = walking days (days/week) × walking minutes (minutes/day). All participants were instructed to maintain their usual dietary habits before the assessment of dietary intake.

#### 2.2.2. Measurement of sarcopenia and obesity

Licensed technicians measured muscle mass and body composition using dual X-ray absorptiometry (DXA; Discovery QDR 4,500 W, Hologic Inc., Belford, MA, USA). Participants fasted prior to the assessment and were in the supine position during the assessment. All non-fat and non-bone tissues were assumed to be skeletal muscle. Appendicular skeletal muscle mass (ASM) was calculated as the sum of skeletal muscle mass in both arms and legs, as measured by DXA. The subjects' skeletal muscle mass index (SMI) was calculated as their ASM (kg) divided by their height in meters squared (m^2^). Sarcopenia was defined as SMI values < 7.0 kg/(m^2^) for men and < 5.4 kg/(m^2^) for women, as recommended by the Asian Working Group for Sarcopenia (AWGS) (30).

Obesity was classified by measuring the WC, which was measured at the narrowest point between the iliac crest and the lower border of the rib cage during expiratory to the closest 0.1 cm. Obesity was defined as a WC > 90 cm in men and > 85 cm in women (31).

#### 2.2.3. Assessment of coffee and nutrient intake

Daily food intake was measured using the 24 h recall method, and daily nutrient intake was calculated using Can-Pro 2.0, a nutrient intake assessment software developed by the Korean Nutrition Society (Seoul, Korea). Information about the frequency of coffee consumption and macronutrient intake was investigated using a 63-item food frequency questionnaire. Self-reported coffee intake data were obtained from the dietary interviews. Participants were asked to indicate how frequently they consumed coffee daily, and the frequency of coffee intake was categorized into 10 groups: rarely, 6–11 cups per year, 1 cup per month, 2–3 cups per month, 1 cup per week, 2–3 cups per week, 4–6 cups per week, 1 cup per day, 2 cups per day, and ≥ 3 cups per day. The participants were re-categorized into four coffee intake groups as follows: participants who did not drink coffee (< 1 cup/day), participants who drank 1 cup of coffee per day (1 cup), participants who drank 2 cups of coffee per day (2 cups), and participants who drank 3 or more cups of coffee per day (≥ 3 cups). The reason for dividing participants into four groups is that the existing sample was too detailed and the difference between the group data was small. The daily intake of total energy, carbohydrates, proteins, and fat was assessed.

### 2.3. Data analysis

Data were analyzed using SPSS 27.0 Window version (IBM, Armonk, NY, USA). The responses were weighted by reference to a multistage, complex, probability sampling design. Data are expressed as absolute numbers and estimated percentages (with standard errors (SE) or the means ± standard deviation (SD). The χ2 test or Student's *t*-test was used to evaluate the differences in demographic and clinical characteristics between the coffee intake and sarcopenic obesity groups. Multivariate logistic regression analysis was used to investigate the association between coffee intake, sarcopenia, and obesity. Odds ratios (ORs) and 95% confidence intervals (CIs) were estimated using multiple logistic regression analysis. Statistical significance was set at *p* < 0.05.

## 3. Results

**Figure 2:**
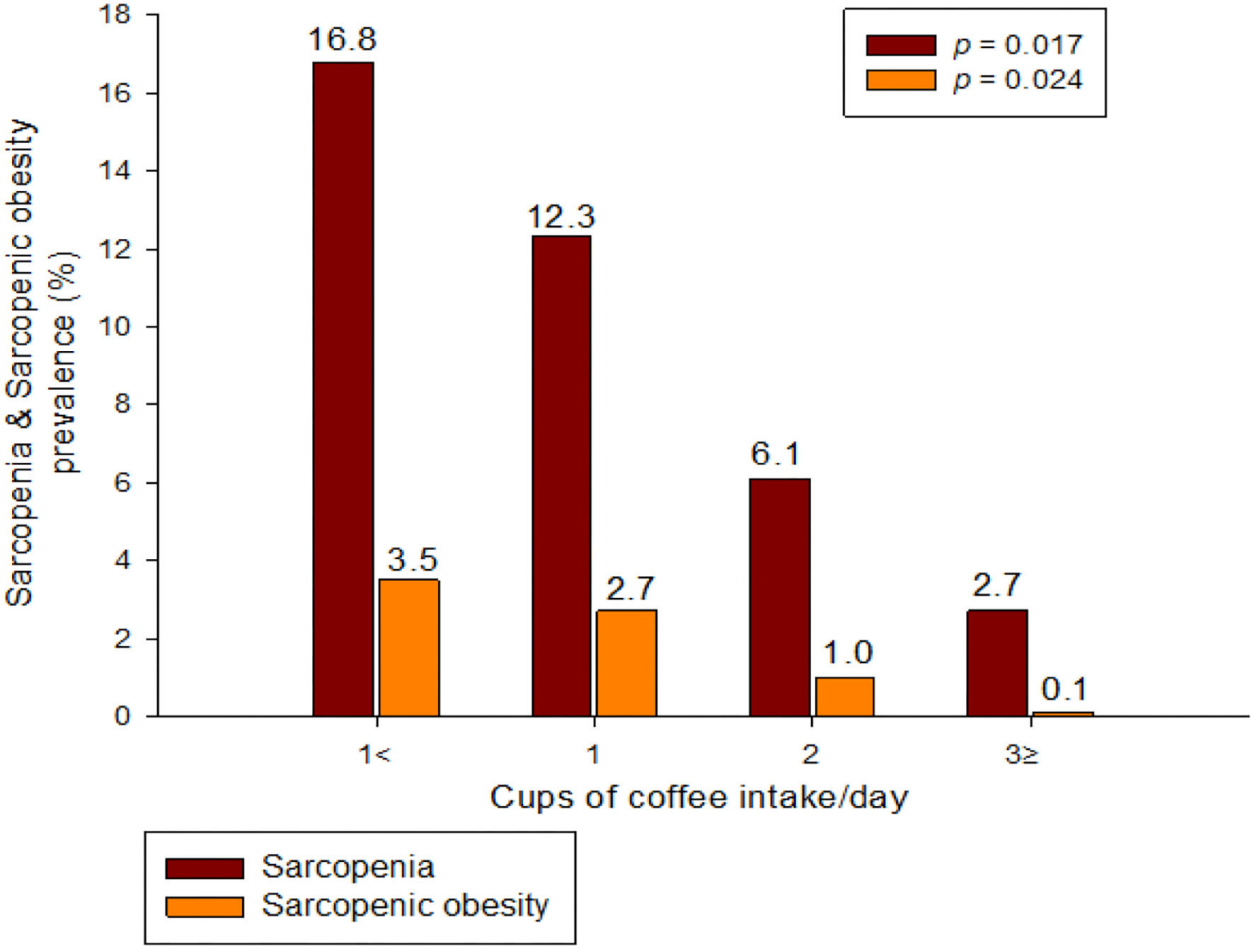
Sarcopenia and sarcopenic obesity prevalence according to coffee intake.

Figure 2 shows the prevalence of sarcopenia and S+O+ according to coffee intake among the elderly population. It can be seen that the higher the amount of coffee consumed in both groups, the more significant the decrease in prevalence rate (Sarcopenia: 16.8, 12.3, 6.1, 2.7%; S+O+: 3.5, 2.7, 1.0, 0.1%).

Table 1 shows the characteristics of the participants according to sarcopenia and obesity status. Triglycerides, fasting glucose, BMI, and total cholesterol were significantly higher in obese individuals (S+O+, S-O+), regardless of whether they had sarcopenia or not. In addition, the proportion of resistance exercise above moderate intensity in the S+O+, S+O-, and S-O+ groups compared to the S-O- group was significantly lower (3.1 vs. 7.4 vs. 7.5 vs. 9.3% in ≥ 4 days/week resistance exercise), and aerobic exercise time was high (65.96 vs. 68.61 vs. 74.25 vs. 55.78 in TWT). The S-O- group had the highest energy intake. There were also significant differences in carbohydrates, proteins, and fats (the three major nutrients), and there was a significant difference in all other groups compared with S-O-.

**Table 1:** Characteristics of subjects stratified by sarcopenia and obesity.

**Variables**	**S+O+ ** **(*n* = 179)**	**S-O+** ** (*n* = 780)**	**S+O-** ** (*n* = 827)**	**S-O-** **(*n* = 875)**	***p*-value**
Age (y)	72.93 ± 0.48^a^	71.27 ± 0.19^b^	72.54 ± 0.21^c^	71.16 ± 0.19^b^	< 0.0001
Height (cm)	155.45 ± 0.79	156.84 ± 0.35	156.63 ± 0.38	157.04 ± 0.37	0.228
Weight (kg)	59.88 ± 0.64^a^	66.23 ± 0.36^b^	51.63 ± 0.31^c^	57.32 ± 0.31^d^	< 0.0001
Systolic BP (mmHg)	132.67 ± 1.35	133.45 ± 0.74	131.50 ± 0.92	130.99 ± 0.80	0.064
Diastolic BP (mmHg)	75.92 ± 0.73	78.10 ± 0.45	76.03 ± 0.47	77.01 ± 0.45	0.226
Waist circumference (cm)	91.20 ± 0.34^a^	93.27 ± 0.23^b^	76.68 ± 0.27^c^	80.58 ± 0.23^d^	< 0.0001
Triglyceride	162.53 ± 8.73^a^	158.70 ± 3.56^a^	140.01 ± 3.66^b^	133.90 ± 3.33^b^	< 0.0001
Fasting glucose (mg/dL)	106.40 ± 2.17^a^	109.18 ± 1.22^a^	100.93 ± 0.87^b^	100.83 ± 0.82^b^	< 0.0001
BMI (kg/m^2^), mean (SD)	24.75 ± 0.17^a^	26.90 ± 0.12^b^	21.01 ± 0.09^c^	23.17 ± 0.09^d^	< 0.0001
Total cholesterol	202.74 ± 3.39^a^	197.33 ± 1.41^a^	191.63 ± 1.49^b^	189.80 ± 1.27^b^	< 0.0001
HDL-C	45.33 ± 1.09^ab^	43.70 ± 0.40^b^	47.49 ± 0.50^a^	46.60 ± 0.48^a^	< 0.0001
Marital status, (%) (living with spouse)	54.2	63.5	65.1	70.6	0.003
**Individual income**					
Q1 (Lowest) Q2 Q3 Q4 (Highest)	20.4 25.2 26.3 28.2	23.0 24.5 25.0 27.5	23.7 28.2 23.2 24.8	27.7 22.5 25.6 24.3	0.326
Smoking status (%) (current-/ex-/non-smoker)	22.1/6.6/71.3	21.6/12.7/65.7	35.5/13.3/51.2	34.1/13.2/52.7	< 0.0001
Drinking status (%) (current-/non-drinking)	40.1/59.9	49.5/50.5	51.6/48.4	55.2/44.8	0.011
**Resistance exercise**					0.013
Never	91.4	86.5	86.4	81.1	
1–3 days/wk	5.5	6.1	6.2	9.6	
≧ 4 days/wk	3.1	7.4	7.5	9.3	
Aerobic exercise (TWT)	65.96 ± 7.66^a^	68.61 ± 4.18^a^	74.25 ± 3.76^a^	55.78 ± 3.47^b^	0.001
Energy intake (kcal)	1,506.99 ± 41.89^a^	1,649.71 ± 33.36^b^	1,558.56 ± 26.32^a^	1,763.83 ± 23.02^c^	< 0.0001
Carbohydrate intake (g)	278.40 ± 7.24^a^	304.87 ± 6.34^b^	287.95 ± 4.66^a^	319.46 ± 4.58^b^	< 0.0001
Protein intake (g)	47.73 ± 1.78^a^	53.75 ± 1.32^b^	50.42 ± 1.25^ab^	57.11 ± 1.11^c^	< 0.0001
Fat intake (g)	20.02 ± 1.35^ab^	21.90 ± 0.73^ab^	19.78 ± 0.65^a^	23.64 ± 0.77^b^	< 0.0001
ASM (kg)	13.91 ± 0.27^a^	17.20 ± 0.16^b^	14.38 ± 0.13^a^	17.14 ± 0.14^b^	< 0.0001
SMI (kg/(m^2^))	5.68 ± 0.06^a^	6.91 ± 0.04^b^	5.79 ± 0.03^a^	6.85 ± 0.03^b^	< 0.0001

Data were presented as means ± SD or number (%). a, b, c, d the same letters indicate the non-significant difference between groups based on Bonferroni multiple comparison test.

Table 2 shows the characteristics of the participants according to their coffee intake. Age, blood pressure, WC, triglycerides, fasting glucose, BMI, total cholesterol, HDL, and individual income according to coffee intake were not different between groups. As the coffee intake increased, the ratio of high-intensity resistance exercise tended to increase (5.9 vs. 7.2 vs. 10.8 vs. 11.4%). However, there was no significant difference in aerobic exercise between groups. In terms of nutritional intake, total energy, carbohydrate, protein, and fat intake were all significantly higher in the group with an intake of 2 or more cups of coffee than in the group with an intake of 1 or fewer cups.

**Table 2:** Characteristics of subjects stratified by coffee intake.

**Variables**	**Cups of coffee intake**				***p*-value**

	**1**< **(*****n*** = **1,140)**	**1** **(*****n*** = **810)**	**2** **(*****n*** = **465)**	**3** ≧ **(*****n*** = **246)**	
Age (y)	71.96 ± 0.20	71.95 ± 0.21	71.20 ± 0.23	71.15 ± 0.37	0.147
Height (cm)	155.23 ± 0.33^a^	156.38 ± 0.33^b^	158.76 ± 0.48^c^	160.78 ± 0.62^d^	< 0.0001
Weight (kg)	57.29 ± 0.38^a^	58.21 ± 0.41^a^	60.32 ± 0.57^b^	61.16 ± 0.73^b^	< 0.0001
Systolic BP (mmHg)	132.04 ± 0.70	131.30 ± 0.79	132.72 ± 1.07	133.00 ± 1.23	0.713
Diastolic BP (mmHg)	76.95 ± 0.40	76.55 ± 0.40	77.33 ± 0.63	77.65 ± 0.65	0.430
Waist circumference (cm)	83.75 ± 0.35	83.97 ± 0.40	84.69 ± 0.52	84.23 ± 0.68	1.000
Triglyceride	151.25 ± 3.66	145.09 ± 3.77	134.12 ± 4.20	142.22 ± 6.95	0.790
Fasting glucose (mg/dL)	104.19 ± 0.84	103.22 ± 10.2	103.15 ± 1.42	105.31 ± 2.48	1.000
BMI (kg/m2), mean (SD)	23.3 ± 0.12	23.76 ± 0.14	23.89 ± 0.20	23.61 ± 0.25	1.000
Total cholesterol	193.89 ± 1.35	193.40 ± 1.45	192.90 ± 1.94	194.32 ± 2.87	1.000
HDL-C	45.34 ± 0.41	46.41 ± 0.47	46.66 ± 0.64	45.15 ± 0.85	0.363
Marital status, (%) (living with spouse)	61.6	64.4	71.6	75.6	< 0.0001
**Individual income**					
Q1 (Lowest) Q2 Q3 Q4 (Highest)	24.9 24.8 26.5 23.8	24.9 27.7 20.6 26.8	25.8 22.3 26.7 25.2	18.8 22.3 27.6 31.4	0.085
Smoking status (%) (current-/ex-/non-smoker)	23.5/8.6/67.9	26.9/12.2/60.9	35.1/20.5/44.5	58.4/16.2/25.5	< 0.0001
Drinking status (%) (current-/non-drinking)	43.1/56.9	53.1/46.9	62.0/38.0	60.8/39.2	< 0.0001
**Resistance exercise**					0.006
Never	88.3	85.3	79.5	81.2	
1–3 days/wk	5.9	7.4	9.6	7.4	
≧ 4 days/wk	5.9	7.2	10.8	11.4	
Aerobic exercise (TWT)	69.28 ± 3.12	67.09 ± 3.95	59.98 ± 4.76	59.98 ± 6.54	0.603
Energy intake (kcal)	1,581.33 ± 22.95^a^	1,597.40 ± 24.44^a^	1,798.20 ± 38.11^b^	1,823.08 ± 57.42^b^	< 0.0001
Carbohydrate intake (g)	294.35 ± 44.10^a^	294.70 ± 4.20^a^	322.23 ± 6.92^b^	326.06 ± 10.20^b^	0.005
Protein intake (g)	51.38 ± 1.01^a^	51.85 ± 1.17^a^	57.59 ± 1.54^b^	59.14 ± 2.43^b^	0.005
Fat intake (g)	19.17 ± 0.59^a^	20.68 ± 0.69^a^	25.70 ± 1.07^b^	28.39 ± 1.46^b^	< 0.0001
ASM (kg)	15.42 ± 0.14^a^	15.76 ± 0.14^a^	17.02 ± 0.20^b^	18.30 ± 0.27^c^	< 0.0001
SMI (kg/(m^2^))	6.32 ± 0.04^a^	6.36 ± 0.04^a^	6.67 ± 0.05^b^	7.00 ± 0.07^c^	< 0.0001

Data were presented as means ± SD or number (%). a, b, c, d the same letters indicate the non-significant difference between groups based on Bonferroni multiple comparison test.

Table 3 shows the association between coffee intake, sarcopenia, and obesity. In model 4, adjusted for all possible covariates, the association between coffee intake and sarcopenia was 1.871 (95% CI 1.227–2.853) in the intake of < 1 cup of coffee, 1.831 (95% CI 1.223–2.741) in the intake of 1 cup of coffee, and 1.633 (95% CI 1.049–2.544) in the intake of 2 cups of coffee. However, there was no significant difference in the association between coffee intake and obesity among all groups and models.

**Table 3:** Odd ratios for obesity and sarcopenia by coffee intake.

**Model**		**Cups of coffee intake/day odds ratio (95% CI)**			
		**1**<	**1**	**2**	**3** ≧
Sarcopenia	1	1.656 (1.150–2.384)^**^	1.567 (1.087–2.259)^*^	1.250 (0.855–1.829)	Ref.
	2	1.700 (1.157–2.497)^**^	1.597 (1.086–2.347)^*^	1.287 (0.872–1.901)	Ref.
	3	1.825 (1.191–2.796)^**^	1.785 (1.181–2.697)^**^	1.524 (0.979–2.371)	Ref.
	4	1.871 (1.227–2.853)^**^	1.831 (1.223–2.741)^**^	1.633 (1.049–2.544)^*^	Ref.
Obesity	1	1.195 (0.851–1.677)	1.304 (0.916–1.857)	1.210 (0.837–1.749)	Ref.
	2	0.949 (0.672–1.342)	1.064 (0.751–1.508)	1.097 (0.755–1.594)	Ref.
	3	1.453 (0.879–2.404)	1.474 (0.890–2.443)	1.320 (0.757–2.303)	Ref.
	4	1.433 (0.897–2.398)	1.498 (0.897–2.500)	1.315 (0.737–2.348)	Ref.

Model 1: crude.

Model 2: adjusted for age, sex.

Model 3: adjusted for variables in Model 2 + weight, height, triglyceride, fasting glucose, smoking status, drinking status.

Model 4: adjusted for variables in Model 3 + marital status, resistance exercise, aerobic exercise, total cholesterol, HDL-C, energy intake, carbohydrate intake, protein intake, fat intake.

Sarcopenia reference category: non-sarcopenia.

Obesity reference category: non-obesity.

* p < 0.05,

** p < 0.01.

Table 4 shows the association between coffee consumption and sarcopenic obesity. In model 4, there was a significant difference between 5.861 (95% CI 2.024–16.971) in < 1 cup of coffee, 6.245 (95% CI 2.136–18.260) in 1 cup of coffee, and 4.323 (95% CI 1.457–12.824) in 2 cups of coffee intake compared to 3 cups of coffee. In contrast, in the case of sarcopenia or obesity only (S+O- or S-O+), no significant difference was found in any model.

**Table 4:** Odds ratios for sarcopenic obesity by coffee intake.

	**Coffee intake**	**Odds ratio (95% CI)**		

		**S**+**O**+	**S**+**O-**	**S-O**+
Model 1	1 <	7.138 (2.530–20.136)^***^	1.487 (0.979–2.258)	1.166 (0.796–1.706)
	1	7.438 (2.608–21.217)^***^	1.443 (0.950–2.192)	1.265 (0.845–1.894)
	2	4.325 (1.496–12.502)^**^	1.174 (0.741–1.859)	1.154 (0.736–1.808)
	3 ≧	Ref.	Ref.	Ref.
Model 2	1 <	5.412 (1.896–15.445)^**^	1.413 (0.908–2.199)	0.906 (0.609–1.350)
	1	5.785 (2.009–16.656)^**^	1.373 (0.879–2.144)	1.011 (0.673–1.519)
	2	3.985 (1.357–11.701)^*^	1.167 (0.730–1.865)	1.038 (0.658–1.637)
	3 ≧	Ref.	Ref.	Ref.
Model 3	1 <	5.632 (2.001–15.851)^**^	1.555 (0.946–2.557)	1.187 (0.672–2.097)
	1	5.942 (2.063–17.116)^**^	1.446 (0.898–2.330)	1.141 (0.672–1.995)
	2	4.163 (1.412–12.277)^*^	1.333 (0.796–2.2234)	1.126 (0.601–2.108)
	3 ≧	Ref.	Ref.	Ref.
Model 4	1 <	5.861 (2.024–16.971)^**^	1.531 (0.923–2.539)	1.135 (0.630–2.045)
	1	6.245 (2.136–18.260)^**^	1.463 (0.918–2.332)	1.159 (0.648–2.072)
	2	4.323 (1.457–12.824)^**^	1.416 (0.844–2.375)	1.139 (0.587–2.210)
	3 ≧	Ref.	Ref.	Ref.

Model 1: crude.

Model 2: adjusted for age, sex.

Model 3: adjusted for variables in Model 2 + weight, height, triglyceride, fasting glucose, smoking status, drinking status.

Model 4: adjusted for variables in Model 3 + marital status, resistance exercise, aerobic exercise, total cholesterol, HDL-C, energy intake, carbohydrate intake, protein intake, fat intake.

Sarcopenic obesity reference category: S-O- (Non-sarcopenia and non-obesity).

* p < 0.05,

** p < 0.01,

*** p < 0.001.

## 4. Discussion

This study aimed to determine the association between S+O+ and coffee intake. The main result of this study was that S+O+ and coffee intake were independently associated after adjusting for various confounding variables such as age, smoking and drinking, exercise, and nutrition. In addition, there was an association between sarcopenia and coffee intake, but no association with obesity was seen. Moreover, as shown in Figure 2, the prevalence of both sarcopenia and S+O+ significantly decreased as the coffee intake increased (sarcopenia: 16.8, 12.3, 6.1, 2.7%; S+O+: 3.5, 2.7, 1.0, 0.1%).

Coffee intake was significantly associated with S+O- (Figure 2, Table 3). A previous study suggested that habitual coffee consumption is positively related to SMI, and that coffee is effective in preventing muscle mass loss (19). In another study, coffee treatment suppressed a decrease in muscle weight and grip strength in mice and proved that coffee was effective in sarcopenia by activating the regenerative ability of skeletal muscles (20). The results of these previous studies are consistent with those of this study. In this study, it can be seen that the average SMI was ~1.11% higher in the intake of 3 or more cups of coffee than in that of 1 cup of coffee (6.32 vs. 7.00, Table 2). Although this may seem a small difference, the 1.11% difference is quite large, as the decrease in muscle mass with age is reported to be ~0.5% per year (32).

Unlike sarcopenia, obesity was not associated with coffee intake (Table 3). Studies showing that coffee consumption is not related to obesity based on WC or BMI criteria have been supported by several previous studies. In a previous study, there was no association between obesity and WC, BMI, and coffee intake (24). In addition, a longitudinal follow-up study of 15 years by Walk et al. revealed that coffee consumption was not associated with WC (33). In contrast, another study suggested that the higher the consumption, the higher the risk of obesity (34, 35). However, contrary to this study, in the case of these previous studies, it seems that it appeared contrary to the results of this study because the association between coffee containing sugar-sweetened or non-dairy creamer and obesity was analyzed in these studies.

Several explanations are possible for the mechanisms underlying the association between sarcopenia, obesity, and coffee intake. First, coffee contains chemical components, such as caffeine, diterpenes, chlorogenic acid, and polyphenol, which have anti-inflammatory and antioxidant effects (36, 37). Chemicals with these properties can play a role in preventing sarcopenia by inducing autophagy, which is essential for the proper regeneration of mitochondria and maintenance of muscle mass (38, 39). In a previous study, 4 weeks of coffee intake in elderly mice significantly improved muscle strength, muscle mass, inflammatory index, and regenerative capacity compared with the control group (20). In addition, chronic consumption of coffee in female mice induced autophagy in the skeletal muscle, heart, and liver (37), and polyphenol, the main antioxidant component, also stimulates autophagy (40). Therefore, it is thought that the chemical components of coffee that perform anti-inflammatory and antioxidant activities induce autophagy to reduce muscle homeostasis and oxidative stress in the mitochondria during aging, thereby reducing the risk of S+O+.

Second, coffee intake may increase the number of satellite cells that enable muscle regeneration and improve skeletal muscle hypertrophy (20, 41). A previous study has shown that coffee increases skeletal muscle satellite cells in aged mice, thereby promoting regeneration of skeletal muscles damaged by aging (20). In addition, dietary coffee supplementation increases muscle function and skeletal muscle hypertrophy due to a decrease in myostatin and an increase in insulin growth factor expression (41). For these reasons, it is judged that the intake of coffee prevents muscle loss and weakening.

Third, habitual consumption caffeine such as coffee is effective in increasing physical activity (42). In several previous studies, high caffeine consumption was independently associated with improved physical performance (43), and these physical functions appear as fast gait speed, reduced risk of falls, and improved attention in the elderly (18, 44, 45). Moreover, high-capacity caffeine clearly influences a wide range of physical performance indicators such as muscle strength, endurance, and high-intensity resistance exercise (42, 46, 47). A difference in physical activity according to coffee intake was also observed in the results of this study. The rate of resistance exercises more than 4 days a week was higher in the group with higher coffee intake (5.9 vs. 7.2 vs. 10.8 vs. 11.4 in Table 2). Therefore, coffee intake may effectively offset physical weakness.

As shown in Table 4, single diseases, such as S+O- or S-O+, did not show any correlation with coffee intake, but a significant association was found in S+O+. In Model 4, which adjusted the influencing factors, S+O+ was 5.861 (95% CI 2.024–16.971) in the intake of <1 cup of coffee compared to the intake of 3 or more cups of coffee. Consumption of many caffeine improves physical performance (43, 48), and combinations of coffee and exercise enhances fat oxidation (49). It is thought that this result was caused by the increased fat acid β-oxidation, which prevented the accumulation of lipids by reducing oxidation stress and inflammation (50). In addition, improved physical activity and adequate nutrition lead to increased muscle mass and muscle strength (51, 52). According to the results of this study, the group of 2 and 3 or more cups of coffee had a higher intake of carbohydrates, proteins, and fats, and a higher rate of high-intensity resistance exercise compared to the group of 1 and <1 cup of coffee. As such, high-intensity resistance exercise due to caffeine intake and adequate nutrition are thought to not only increase muscle strength and muscle mass but also promote fat oxidation, resulting in body fat reduction (53–55). As a result, the proportion of S+O+ in the group that consumed less coffee increased.

To summarize the results of this study, the rate of S+O+ in the elderly who consumed <2 cups of coffee was significantly higher than that of the elderly who consumed more than 3 cups of coffee per day. In addition, coffee intake was associated with sarcopenia but not with obesity. Therefore, it suggests that coffee intake is possible to increase the amount of physical activity and prevent musculoskeletal loss in these patients.

Despite its meaningful findings, there are several limitations in evaluating the results of this study. First, when evaluating sarcopenia in this study, various physical performance such as grip strength and gait speed were not evaluated. However, according to several previous studies, appropriate muscle mass and lean mass are used as variables that can represent physical performance (56–58). Second, this study did not accurately measure the content of caffeine consumed and did not completely exclude the possibility of the interference of other substances that can supply antioxidants such as green tea, milk, vegetables, and fruits. Third, subjects who participated in the KNHANES may have affected the outcome analysis due to the lack of participation of a small number of severely sarcopenia or obese patients. However, because the data were obtained from the national population, it is not expected that the disturbance factor for a small number of people will have a significant impact on the results. Fourth, although this study may help provide more information about the nature of the relationship, it was a cross-sectional study that measured both S+O+ and coffee intake at the same time. Consequently, it was not possible to determine the temporal relationship between coffee intake and S+O+, and it was impossible to pinpoint the order of the fundamental causes between the two factors. As a result, care should be taken when interpreting the results. Therefore, it would be worthwhile to reveal a mechanism that can clarify the causal association between S+O+ and coffee intake through future longitudinal studies.

## 5. Conclusion

This study was conducted to determine the association between sarcopenic obesity and coffee intake in elderly Koreans. The rate of sarcopenic obesity was higher in the elderly who consumed less than one cup of coffee compared to the elderly who consumed more than three cups of coffee a day. Moreover, there was a significant association between coffee intake and sarcopenia, but not obesity.

## Data availability statement

Publicly available datasets were analyzed in this study. This data can be found at: https://knhanes.kdca.go.kr/knhanes.

## Author contributions

D-YL and SS conceived of the study and participated in its design and coordination, drafted the manuscript, and wrote the manuscript. SS drafted the proposal for this project. D-YL contributed to sampling, data collection, and statistical analysis. All authors have read and approved the final manuscript.
